# Editorial: Progress in Translational Neuroimaging: Integrating Pathways, Systems, and Phenomenology in Neurology and Psychiatry

**DOI:** 10.3389/fpsyt.2020.00682

**Published:** 2020-07-10

**Authors:** Drozdstoy Stoyanov, Paolo Brambilla

**Affiliations:** ^1^ Department of Psychiatry and Medical Psychology and Research Institute, Plovdiv Medical University, Plovdiv, Bulgaria; ^2^ Department of Neurosciences and Mental Health, Fondazione IRCCS Ca' Granda Ospedale Maggiore Policlinico, Milan, Italy; ^3^ Department of Pathophysiology and Transplantation, University of Milan, Milan, Italy

**Keywords:** neuroimage analysis, phenomenology, diagnosis, psychiatry, nosology and classification of mental disorders

Classification is taxonomic nomenclature system established for the purposes of statistical analysis of the phenomena and in order to facilitate and uniform the language of professionals in different countries. Classification systems in medicine, hence in psychiatry are composed of nosological units. The latter as well as the relevant methods for their exploration are characterized with the criteria of validity, reliability, specificity, and sensitivity.

Whenever we introduce some term, method or assessment system, we should inquire whether it can capture the exact phenomenon *it is intended* to capture. In other words, when we introduce a term like “depression” or “depressiveness” as a symptom and develop relevant instruments to measure it (inventories and clinical interviews), we are supposed to ascertain that it measures specifically depression and not some other related, phenomenon, e.g. delusions. When two different assessment tools for depression happen to coincide in their score/interpretation, it is regarded as *convergent validity*, and whenever their results are discrepant it is considered as divergent or *discriminative validity*. Divergent validity should generally characterize phenomena which differ each from other and in this respect is supposed to assist differential diagnosis between them. In this context, a valid measure (e.g. test) for depression should have convergent validity with other test, established to assess depression and discriminative validity with tests, designed for assessment of paranoid delusions. In other fields of medicine, external criteria for validity are introduced, such as biological measures. No such criteria have been introduced so far in clinical psychiatry.

Psychiatric diagnosis is characterized with *relatively high level of reliability* (repeated clinical ratings from different clinicians) and problematic *validity* (lack of commonly used biological markers to reify the diagnosis).

Over the past two decades there have been established mainly post-hoc correlations between the different types of clinical tools (cognitive assessment tests, interviews, and inventories) on one hand and imaging neuroscience. This far different kinds of measures represent mere statistical correlations with limited or no reference to the mechanism of disorder and therefore cannot be effectively translated and embodied into normative criteria, diagnostic standards, and clinical evaluation procedures (1). This undermines the reification of psychological clinical measures and mental disorders as diagnostic entities (2).

There has been raised a conceptual issue: What is the *subject of reification that procedures of translation may address*? Is it the clinical assessment inventory/test that is reified by means of functional MRI for example or the opposite?

This Research Topic has particular focus on whether and to what extent it is feasible to translate data from multimodal neuroimaging in terms of cross-validation against different trait and state measures, such as the Temperament and Character Inventory or Paranoid-Depressive Scale (Squarcina et al.) and PVSAT/PASAT (Iancheva et al.).

We assume that in this manner there is provided meaningful insight into the relevance of multimodal neuroimaging resources for both explanatory mechanisms of normal mental functions and brain dysfunctions which underlie mental disorders. In this fashion various papers contribute with neuroimaging findings to underpin the clinical measures of and the nosological entities.

In one of the presented papers in this Research Topic (Stoyanov et al.), the most powerful functional MRI pattern which allows discriminative validation of schizophrenia from affective disorders is comprised of BOLD signal corresponding to negative signature on depressive items response and positive signature to neutral items response from interest scale along with the signal associated with paranoid items. Essentially this means that neutral items are not neutral for patients with schizophrenia, but incorporated into their psychotic disturbance system.

In this Research Topic, we tried to consider all these above-mentioned critical issues by integrating studies on diverse pathways, neurosystems and phenomenology in both psychiatric and neurologic disorders. Specifically, 19 articles have been published on a variety of themes, being 11 of them original papers mostly implementing neuro-imaging approaches. Three studies focussed on schizophrenia, showing 1) instability of the default mode network (DMN) and impaired cognitive control when patients experience auditory verbal hallucinations (AVH), and 2‑3) preliminary possibility to disentangle brain activation due to paranoia in schizophrenia and depression by using a self-evaluation scale. Three investigations explored mood disorders, reporting 1) mismatch between cerebral blood supply and oxygen metabolism in bipolar disorder, being a potential brain sign of energy homeostasis inefficiency, 2) no effect of the BDNF genotype on encoding-related neural activity in bipolar patients, with a superior memory performance in Met carriers, and 3) symptom improvement after 3-weeks of repeated transcranial magnetic stimulation (rTMS) therapy on the left dorsolateral prefrontal cortex (5 consecutive weekdays every week) in patients with unipolar depression. Three other reports detected fascinating results in different pathologies suggesting: 1) reduced interhemispheric functional connectivity in patients with obsessive-compulsive disorder (OCD), 2) activation in left Brodmann area (BA) 40 (particularly supramarginal gyrus) as a possible mechanism for diminishing fatigue impact on cognitive functioning in cognitively preserved patients with multiple sclerosis (MS), and 3) brain reorganization due to pre-/perinatal damage in left hemisphere (periventricular and cortico-subcortical lesions).

Interestingly, in healthy subjects the biochemical brain basis (phosphocreatine plus creatine -PCr+Cre-, glycerophosphocholine plus phosphocholine -GPC+PC-, and myo-inositol) of specific personality traits during adolescence were here presented with particular regards to self-directedness and self-transcendence along with the functional brain activations during memory paradigm performance after intensive learning particularly in both occipital and temporal regions.

Three single cases reported intriguing perspectives on delirious mania (due to mild encephalitis with reversible splenial lesion), schizophrenia (potential amelioration of eyeblink conditioning -EBC- after cerebellar transcranial direct current stimulation -tDCS-), and Alzheimer’s disease (PSEN-1 gene mutation in an early-onset patient). Finally, five reviews described the state of the art of cognitive and neuroimaging correlates in PTSD, depression, substance abuse and eating disorders, debating their translational and clinical implications, and future directions whereas.

In conclusion, we believe this is a comprehensive and well composed Research Topic of Frontiers in Psychiatry pointing out key topics for translational psychiatric neurosciences and neuroimaging. In particular, this Research Topic corroborated the fact that nowadays, we need further research on the genetic, neurobiological, and cognitive bases of major psychiatric disorders in order to better delineate different domains charactering commonalities and dissimilarities across different spectrum and continuum diagnostic prototypes (3). This will ultimately help us to have an evolute idea of how human brain impairments affect neuropsychological functions, behavioral characteristics, general functioning, outcome, and disease trajectory of our patients (4). We finally, last but not least, thank the estimated authors who contributed to the issue to allow us to have the privilege to coordinate and guest edit such a prestigious issue.

## Author Contributions

Both authors have contributed on equal basis to this paper.

## Conflict of Interest

The authors declare that the research was conducted in the absence of any commercial or financial relationships that could be construed as a potential conflict of interest.
